# Genotoxic Evaluation of Mexican Welders Occupationally Exposed to Welding-Fumes Using the Micronucleus Test on Exfoliated Oral Mucosa Cells: A Cross-Sectional, Case-Control Study

**DOI:** 10.1371/journal.pone.0131548

**Published:** 2015-08-05

**Authors:** Ana Cecilia Jara-Ettinger, Juan Carlos López-Tavera, María Guadalupe Zavala-Cerna, Olivia Torres-Bugarín

**Affiliations:** 1 Faculty of medicine, Universidad Autónoma de Guadalajara, Zapopan, Jalisco, Mexico; 2 Faculty of medicine, International Program, Universidad Autónoma de Guadalajara, Zapopan, Jalisco, Mexico; University of Science and Technology of China, CHINA

## Abstract

**Background:**

An estimated 800,000 people worldwide are occupationally exposed to welding-fumes. Previous studies show that the exposure to such fumes is associated with damage to genetic material and increased cancer risk. In this study, we evaluate the genotoxic effect of welding-fumes using the Micronucleus Test on oral mucosa cells of Mexican welders.

**Material and Methods:**

We conducted a cross-sectional, matched case-control study of n = 66 (33 exposed welders, and 33 healthy controls). Buccal mucosa smears were collected and stained with acridine orange, observed under 100x optical amplification with a fluorescence lamp, and a single-blinded observer counted the number of micronuclei and other nuclear abnormalities per 2,000 observed cells. We compared the frequencies of micronuclei and other nuclear abnormalities, and fitted generalised linear models to investigate the interactions between nuclear abnormalities and the exposure to welding-fumes, while controlling for smoking and age.

**Results:**

Binucleated cells and condensed-chromatin cells showed statistically significant differences between cases and controls. The frequency of micronuclei and the rest of nuclear abnormalities (lobed-nuclei, pyknosis, karyolysis, and karyorrhexis) did not differ significantly between the groups. After adjusting for smoking, the regression results showed that the occurrence of binucleated cells could be predicted by the exposure to welding-fumes plus the presence of tobacco consumption; for the condensed-chromatin cells, our model showed that the exposure to welding-fumes is the only reliable predictor.

**Conclusions:**

Our findings suggest that Mexican welders who are occupationally exposed to welding-fumes have increased counts of binucleated and condensed-chromatin cells. Nevertheless, the frequencies of micronuclei and the rest of nuclear abnormalities did not differ between cases and controls. Further studies should shed more light on this subject.

## Introduction

### Welding-fume exposure and DNA damage

Approximately, 800,000 people worldwide are occupationally exposed to metal-welding-fumes, this increases the generation of reactive oxygen species (ROS) that damage the genetic material [1]. Evidence suggests that DNA damage (genotoxicity), caused by welding-fumes, plays an important role in the development of lung cancer, which incidence is higher in the welder sub-population compared to general population [1–3].

Iron is the most used metal in Mexican industry; the exposure to iron smokes relates to the initiation, growth, and metastasis of malignant neoplasms [1,4], probably because it is a strong oxidising agent [5]. Aluminium is the second most used metal in the welding industry, and occupational exposure to its fumes is associated with generation of ROS that triggers a cascade of immune responses that lead to lysosomal membrane damage, genotoxicity, and finally, carcinogenesis or cell-death [6–8]. The use of carbon steel (an iron-carbon alloy, with traces of other elements) is also common in Mexican industry; when welded, carbon steel releases fumes that contain iron, molybdenum, copper, chromium, nickel and manganese; the inhalation of these is reported to induce genotoxicity trough ROS generation aforementioned. [1,3–5,7,9,10].

In response to the occupational health risks of the welding industry, the Official Mexican Norm–NOM027 *recommends* certain safety procedures in welding activities, which include:

- To be considered *breathable*, oxygen concentration in atmospheric air should range from 19.5% to 23.5%
- Welders should wear welding face-shields or welding safety-glasses, welding gloves and safety-shoes.
- Welding safety equipment should be safety-checked at least once every month.

As mentioned above, in Mexico, these are *non-mandatory* guidelines [11]. There are no welding training courses available to inform the welders about the risks implied in this profession, and most of them do not wear adequate safety gear nor follow recommended safety procedures. Therefore, the Mexican welder population should be considered vulnerable to occupational health risks related to the welding-industry. This justifies the evaluation of the genotoxic effect of welding-fumes using tests that have proven their reliability for genotoxicity and cancer risk assessment, like the Micronucleus Test [11–13].

### The Micronucleus Test

The Micronucleus Test (MN Test) is an assay used for screening potential genotoxic agents by detecting micronuclei (MNi) and other nuclear abnormalities (NA)–binucleated (BN), lobed-nuclei (LN), condensed chromatin (CC), pyknotic (PK), karyolytic (KL) and karyorrhectic (KR) cells–in the cytoplasm of interphase cells [11,14,15].

The MN Test has been proven repeatedly as a reliable biomarker for genotoxicity [6,13,15–19], cellular instability [20–23], and cell-death [5,6,20,24], and cancer risk [12,22,25,26],

Although the exact molecular basis underlying to the formation of MN and NA remains mostly unknown, there are some explanations about it [27]. MNi may originate from acentric chromosome fragments (or whole chromosomes) unable to migrate to the cell poles during the anaphase [6,14,21]. BN cells contain two nuclei, possibly due to blocking of cytokinesis or cell fusion [28]. Mechanisms of LN cell development seem to involve alterations in the expression of nuclear laminae proteins [29,30]. PK is the hyper-condensation of the nuclei of a dying cell [24], and is an indicator of cell-death as well as CC, KL (ghost-like cell) and KR [15,16].

Previous studies have shown the influence of variables other than genotoxic agents on the frequency of MNi and NA[31], which account for inter-observer and inter-laboratory variability[26], staining methods[32], age and gender of the evaluated individual [33], and genetic polymorphisms [34]. Thus, these factors should be taken into account when conducting MN Test studies.

### Objectives

We hypothesised that the exposure to welding-fumes is positively correlated to the frequency of MNi and NA; we tested the null hypothesis that MNi and NA frequencies were no significantly different between the exposed group and the unexposed control group.

If we could reject the null hypothesis, this study would strengthen the available evidence stating that the exposure to welding-fumes is associated with increased MNi and NA frequencies, and subsequent elevated cancer risk.

## Material and Methods

### Ethics Statement

The Human Research Ethics Committee at Universidad Autónoma de Guadalajara approved this study.

After a clear verbal explanation of the purposes, risks, and benefits from enrolling in the study, we obtained written informed consent from all research participants.

We followed the Mexican laws for health related research projects on humans, established in PROY-NOM-012-SSA3-2007 (DOF: 05-11-2009). In addition, the study met all the points stated in the Federal Law on Protection of Personal Data (DOF: 05-07-2010).

### Study Design

We conducted a cross-sectional, matched case-control study of n = 66. Participants were 33 metal inert gas welders (cases) and 33 matched controls (control:case ratio = 1) recruited in Morelia and Guadalajara, Mexico. Controls were defined as individuals which had never worked in the welding industry.

The inclusion criteria were to have worked as metal inert gas welders for at least 1 year (for cases), and to have signed the informed consent to participate in the study. We excluded individuals which had history of recreational drug use, took any medicines, or had any acute or chronic disease.

Cases and controls were matched for gender, age, and body mass index (BMI); other confounding variables like tobacco use, alcohol consumption, and the presence of dental crowns were imperfectly matched.

Exposure to welding-fumes (1 for cases, 0 for controls), gender, smoking, drinking, and dental crowns were coded as binary variables (1 for presence, 0 for absence); age, BMI, exposure years, and MN and NA counts were coded as continuous variables.

### Sampling and Sample Processing

We obtained exfoliated epithelial cells from the participants' buccal mucosa by smearing the inside lining of the subjects' cheeks with a moistened wooden tongue-depressor. The smears were directly transferred to slides, and dried at room temperature. Afterwards, they were fixed in an 80% ethanol solution during 48 hours, and stained with acridine orange. The samples were stored in an opaque box to keep them free from dirt and to prevent light exposure until they were observed under the microscope. Every slide was coded before observation, so the observer was blinded to the origin of the sample at the moment of microscope observation.

**Fig 1:**
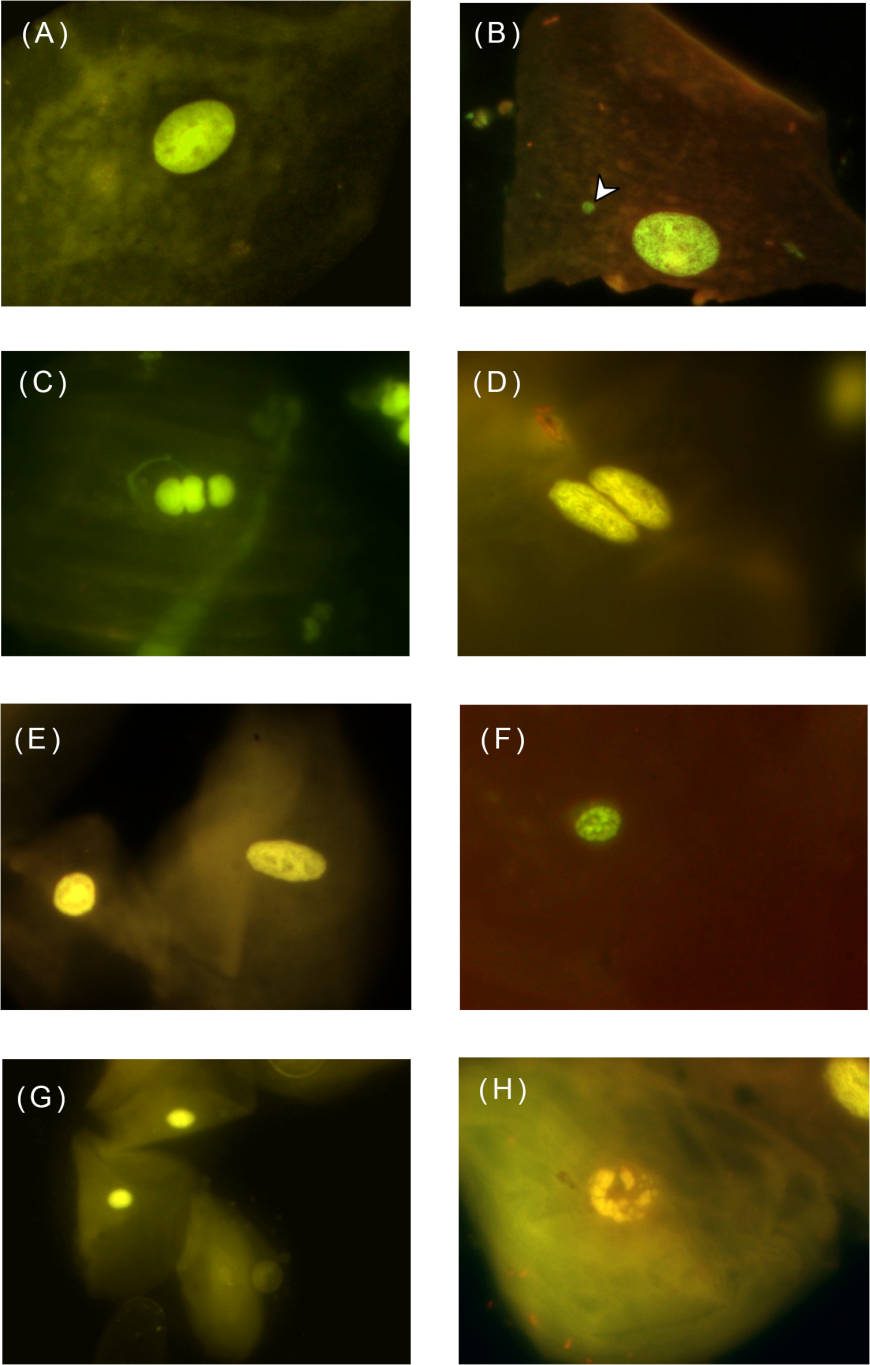
Microphotographs of MNi and NA, identified according to the HUMNxl scoring criteria. The figure shows a series of 8 microphotographs of oral mucosa cells stained with acridine orange at100x optic amplification with a Carl Zeiss IVFL Axiostar Plus microscope, 450–490 nm fluorescence filters. (A) Abnormal buccal cell without any MNi or NA, (B) A buccal cell with the presence of micronucleus(white arrow), (C) a lobed-nuclei cell, (D) a binucleated cell, (E) a pyknotic cell, (F) a condensed-chromatin cell, (G) a karyolytic cell karyorrhectic cell, and (H) karyorrhectic cell.

An experienced reader, blind to the group to which samples belonged, observed the slides under 100x optic amplification with a Carl Zeiss IVFL Axiostar Plus microscope equipped with 450–490 nm fluorescence filters. Using the HUMNxl scoring criteria [35], she identified and counted the number of MNi and NA occurrences per 2000 observed cells per slide (Fig 1).

### Data analysis

For descriptive purposes, age, BMI and exposure years are expressed as mean (standard deviation); gender, smoking, drinking and presence of dental crowns are expressed as percentages; MN and NA counts are expressed as median (Interquartile range (IQR)) and reported as number of occurrences per 2000 cells.

When comparing the mean differences of non-MN Test variables between groups, we did a Welch's two sample t-test for age and BMI, and a Fisher's Exact Test for the non-normally distributed binary data (smoke, drink, dental crowns).

Except for age and BMI, the distribution of the variables was non-normal (Shapiro-Wilks test of normality, p >0.1) [36], and were over-dispersed (variance > mean). Bearing that in mind, we used square root transformation to stabilise the variance, and then proceeded to analyse the data.

We performed a paired Wilcoxon signed rank test with continuity correction to compare the counts of MNi and each NA between cases and controls. Latter, we would fit a quasi-Poisson model, with the MN and NA that showed significant differences as response variables, taking the exposure to welding-fumes as predictor and controlling for confounding variables.

All hypotheses were tested at 95% confidence interval (CI), two-sided, and the level of type I error was set at α = 0.05 (except for the Shapiro-Wilk test, which was α = 0.1)

All the data analyses were done using R Studio with R version 3.1.2 (2014-10-31)–“Pumpkin Helmet” for Linux (64-bit) [37]. Descriptive statistics were performed using the `describe`function from the `psych`package [38]. Squared root transformation was performed using the `sqrt`function from the `base`package. The tests were performed calling the `shapiro.test`, `t.test`and `fisher.test`and `wilcox.test`functions from the `stats`package. Plots were generated using the `sjp.glm`and `ggplot`functions from the `sjPlot`[39] and `ggplot2`[40] packages, respectively.

The data set, and the R scripts for cleaning, exploring and analysing it are available in the fig**share** repository.

## Results

### Descriptive statistics

The mean age of the participants was 40.27 ± 13.62 years, ranging from 18 to 71 years, and the mean BMI was 26.08 ± 3.49 kg/m^2^, which falls in the category of overweight (BMI >24 kg/m^2^), a common health problem in Mexico [41]. The very low presence of women in the workforce of the welding industry in Mexico made impossible for us to find female welders, therefore all of the participants were males.

Because of matching, our data had similar age, BMI, smoking, drinking and dental crowns values, and we didn't observe any statistically significant difference between cases and controls using the Welch's two sample t- tests and the Fisher's Exact Tests (Table 1).

**Table 1:** Descriptive statistics of the sample (n = 66): 33 cases and 33 controls.

Variable	Sample values	Cases	Controls	Test statistic
Dental crowns (percent %)	22.73%	33.33%	12.12%	OR = 3.555 (p = 0.0759)
Tobacco use (percent %)	33.33%	42.42%	24.24%	OR = 2.273 (p = 0.1912)
BMI (mean ± SD kg/m²)	26.08 ± 3.49	25.63 ± 3.37	26.53 ± 3.6	T = 1.0399 (p = 0.302)
Age (mean ± SD years)	40.27 ± 13.62	39.39 ± 13.55	41.15 ± 13.84	t = 0.5211 (p = 0.604)
Alcohol consumption (percent %)	42.42%	42.42%	42.42%	OR = 1 (p = 1)

None of the differences between groups were statistically significant. Welch's Two Sample t-test for unequal variances was used to compare age and BMI difference means between cases and controls (two-sided, 95% confidence interval (CI)). Fisher's exact test for count data was used to compare tobacco use, alcohol consumption and dental crowns (two sided, 95% CI). All p-values are rounded to the third decimal place; a p-value <0.5 would have been considered as significant.

The welders' average exposure years was 16.39 ± 14.04, ranging from 1 to 50 years, and, as it would be expected, the number of exposure years were positively correlated to the age of the age of the cases (Pearson's moment-product correlation = 0.8343, p < 0.001).

### Micronucleus test results

As summarised in Table 2, the MNi median count was 1 (range = 4) per 2000 cells, and did not differ between cases and controls (p = 0.293). For binucleated cells there was a strong and significant difference between cases and controls (p = 0.0014); the difference of condensed-chromatin cells was significant too (p = 0.041). The rest of the nuclear abnormalities were similar between groups, and did not have any significant differences.

**Table 2:** Median (IQR) of the count of micronuclei and other nuclear abnormalities.

	Sample (n = 66)	Cases (n = 33)	Controls (n = 33)	Test statistic (p-value)
Binucleated cells	3.5 (5.5)	8 (6)	3 (2)	V = 144.5 (p < 0.001) ***
Condensed-chromatin cells	3 (4)	4 (2)	0 (3)	V = 382 (p < 0.001) ***
Lobed-nuclei cells	3 (2.75)	3 (3)	3 (3)	V = 201 (p = 0.145)
Karyolyitic cells	3 (1.75)	4 (1)	3 (2)	V = 209.5 (p = 0.3923)
Karyorrhectic cells	0 (1.75)	0 (1)	1 (2)	V = 127.5 (p = 0.519)
Pyknotic cells	0 (0.75)	0 (0)	0 (0)	V = 42 (p = 0.822)
Micronucleated cells	1 (2)	1 (2)	1 (2)	V = 165.5 (p = 0.945)

Values are expressed in number of occurrences per 2000 cells. Median counts were compared using a two-sided Wilcoxon signed rank test with continuity correction. At a 95% CI, p-values <0.05 were considered as significant. Significance codes: <0.001 ‘***’

Then, we selected binucleated and condensed-chromatin cells variables to further investigate their interactions with the exposure to welding-fumes, and to control for confounding variables like BMI, age, exposure years, smoking, drinking and/or presence of dental crowns.

We fitted two quasi-Poisson generalised linear models (GLMs) with the square root link function; we did forward stepwise selection to add the appropriate terms to the initial models:

→BN∼exposure

→(II)CC∼exposure

We compared the initial models to the models that resulted of adding each term using F-tests (ANOVA II); we selected the term that was previously added to the model with the greatest F-value, one term at a time, until the model could not be improved anymore. The final models had the following formulae, that were fed to the `glm`function in R:

→(I)BN∼exposure+tobaccouse

→(II)CC∼exposure

where response variables BN (I) and CC (II) are the counts of binucleated and condensed-chromatin cells per 2000 cells, respectively. Predictors are: “exposure” (1 = exposed | 0 = control), that indicates if the data point corresponds to the exposed group or not; and “tobacco use” stands for the smoking habit (1 = positive | 0 = negative).

**Fig 2:**
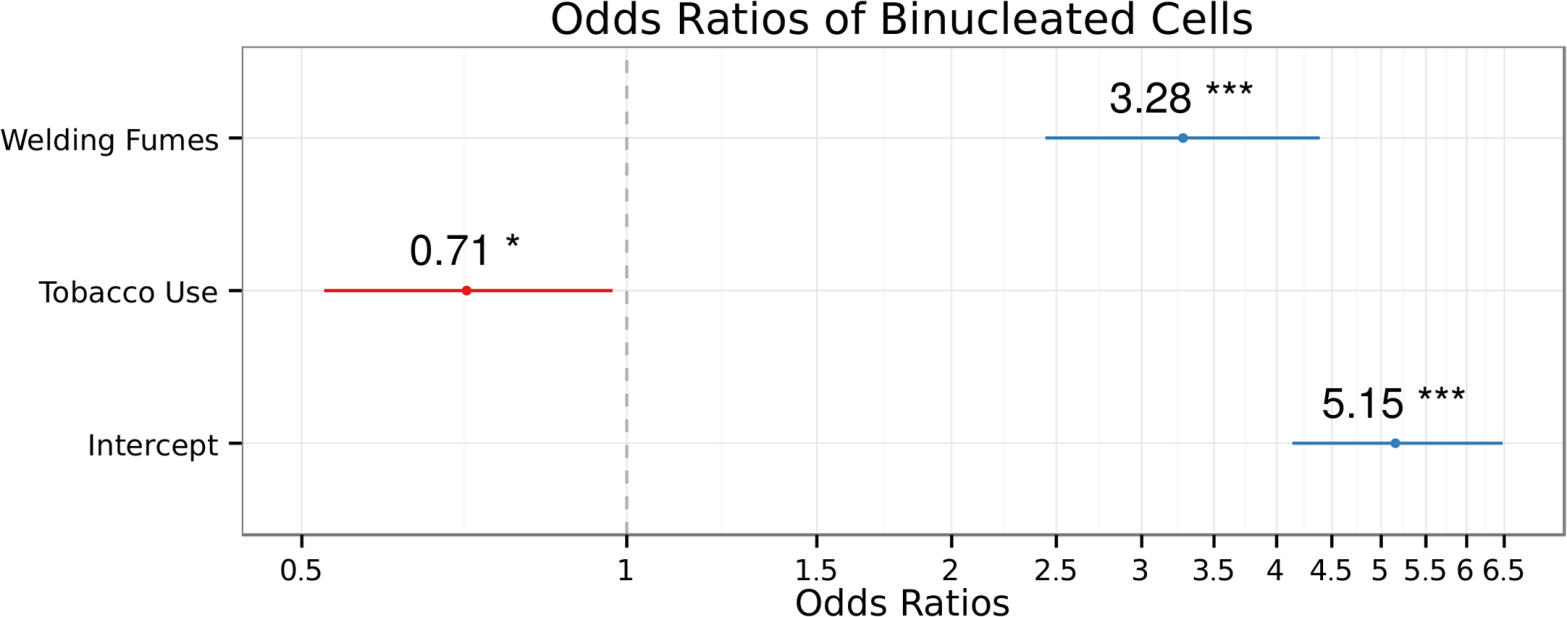
The estimated Odds Ratios of Binucleated Cells depending of the exposure to welding-fumes and tobacco use. BN ~ exposure + tobacco use + intercept. The error bars show the 95% confidence intervals of OR of each term. Intercept = 1.64; R^2^_CS_ = 0.751; R^2^_n_ = 0.792. Significance codes: 0 ‘***’, 0.01 ‘*’.

The results of the BN model (Fig 2 and Table 3) reveal that welders were 1.19 times more likely to have increased BN counts (β = 0.981, p = 2.39 × 10^−6^). Age was a modest, yet significant, predictor of BN (OR = 1.02, beta = 0.0166 p = 0.0068); while participants who smoked, surprisingly showed smaller probabilities of having higher counts of BN cells (OR = 0.54, beta = -0.623, p = 0.0016).

**Table 3:** Results of the binucleated cells quasi-Poisson GLM.

Terms	Estimate	SE	t-value	p-value
(Intercept)	1.6399	0.1123	14.607	< 2× 10^−16^ ***
Case	1.1869	0.1517	7.823	7.29 × 10^−11^ ***
Tobacco use	-0.3415	0.1609	-2.122	0.038 *

SE = Standard Error. Dispersion parameter for quasi-Poisson family = 1.462755; null deviance = 196.24 on 65 degrees of freedom (df); residual deviance = 104.37 on 63 df. Number of Fisher Scoring iterations: 6. At a 95% CI, p-values <0.05 were considered as significant. Significance codes: <0.001 ‘***’, 0.01 ‘*’.

**Fig 3:**
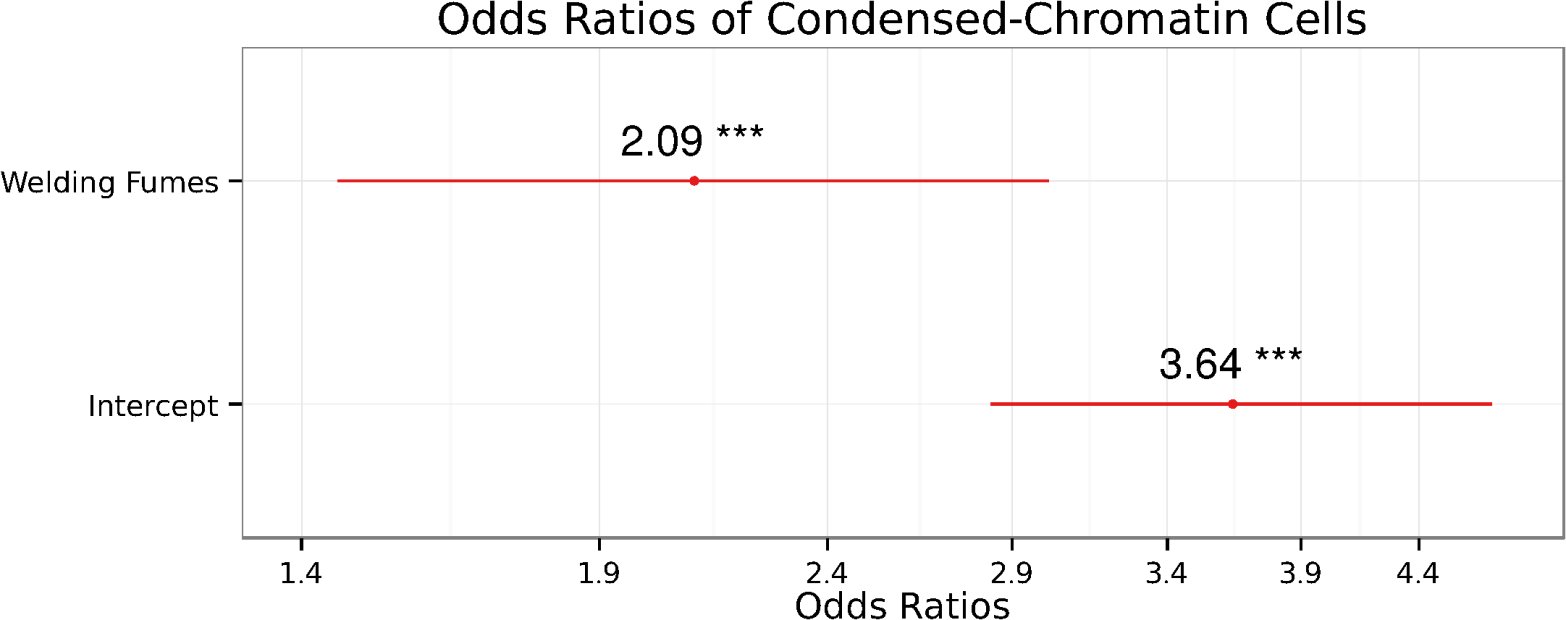
The estimated Odds Ratios of Condensed-Chromatin Cells depending of the exposure to welding-fumes. CC ~ exposure + intercept. The error bars show the 95% confidence intervals of OR of each quasi-Poisson GLM term. Intercept = 1.291; R^2^_CS_ = 0.416; R^2^_n_ = 0.437. Significance codes: <0.001 ‘***’.

The regression analysis of the CC model (Fig 3 and Table 4), shows that welders from our sample had 1.64 times more chance of having significantly more CC cells than their non-exposed counterparts (β = 0.492, p = 0.046). According to our model, the increased frequency of CC cells could only be explained by the exposure to welding fumes.

**Table 4:** Results of the condensed-chromatin cells quasi-Poisson GLM.

Terms	Estimate	SE	t-value	p-value
(Intercept)	1.291	0.1316	9.811	2.23 × 10^⁻14^ ***
Case	0.7391	0.1861	3.971	0.000184 ***

SE = Standard Error. Dispersion parameter for quasi-Poisson family = 2.285754; null deviance = 198.55 on 65 df; residual deviance = 163.09 on 64 df. Number of Fisher Scoring iterations: 5. At a 95% CI, p-values <0.05 were considered as significant. Significance codes: <0.001 ‘***’.

## Discussion

In this study, we aimed to assess the genotoxic effect of welding-fumes on 33 exposed Mexican welders using the Micronucleus Test, and to compare the frequency of observed MNi and NA to the frequencies observed in a matched group of 33 healthy unexposed individuals.

The MNi counts did not differ between cases and controls, yet we found statistically significant differences in binucleated cells (p < 0.001) and condensed-chromatin cells (p < 0.001).

Our findings are consistent with Wultsch et al, who did not report significant differences in the frequency of MNi, but found higher frequencies of BN and CC, and, unlike us, they also found significative differences in LN, KR and KL frequencies [16]. They also measured the blood levels of manganese, molybdenum and nickel, and found that these are associated with higher genetic instability. In his thesis held in Colombia, Tejedor also reported a significant increase of BN cells in welders, but did not find differences in MN cells nor the rest of nuclear abnormalities between cases and controls [42].

Our results contradict the findings of similar studies previously conducted in India, where researchers monitored the genotoxicity of welding-fumes in Indian welders using the MN Test on exfoliated buccal cells also, and found increased MNi counts in the exposed groups [10,43]. Danadevi et al. [9] conducted a similar study, where instead of performing the MN Test on buccal mucosa, they did on human lymphocytes, also found higher MNi frequencies in welders.

However, Danadevi et al and Sudha et al did not use DNA-specific staining methods, which could give false positives as a result, as Nerseyan pointed out in 2006 [32].

At the end, the findings of our study cannot be generalised due to our small sample size and the high variability of our data; thus, bigger research studies focused on more narrow age groups are needed to state the correlation between exposure welding-fumes and the results of the MN test on Mexican welders. However, our results contribute to strengthen the evidence that welding fumes and age are positively correlated with higher frequencies of nuclear abnormalities, at least of binucleated and condensed-chromatin cells.
